# Spatial activation of ezrin by epidermal growth factor receptor and focal adhesion kinase co-ordinates epithelial cell migration

**DOI:** 10.1098/rsob.210166

**Published:** 2021-08-11

**Authors:** Grace K. Chan, John A. McGrath, Maddy Parsons

**Affiliations:** ^1^ Randall Centre for Cell and Molecular Biophysics, King's College London, Guy's Campus, London SE1 1UL, UK; ^2^ St Johns Institute of Dermatology, King's College London, Guy's Campus, London SE1 9RT, UK

**Keywords:** EGFR, FAK, ezrin, focal adhesion, epithelial cell

## Abstract

Epidermal growth factor receptor (EGFR) plays a critical role in the promotion of epithelial cell proliferation and migration. Previous studies have suggested a cooperative role between EGFR and integrin signalling pathways that enable efficient adhesion and migration but the mechanisms controlling this remain poorly defined. Here, we show that EGFR forms a complex with focal adhesion kinase in epithelial cells. Surprisingly, this complex enhances local Src activity at focal adhesions to promote phosphorylation of the cytoskeletal adaptor protein ezrin at Y478, leading to actomyosin contractility, suppression of focal adhesion dynamics and slower migration. We further demonstrate this regulation of Src is due to the suppression of PTP1B activity. Our data provide new insight into EGF-independent cooperation between EGFR and integrins and suggest transient interactions between these kinases at the leading edge of cells act to spatially control signalling to permit efficient motility.

## Graphical abstract

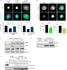

## 1. Introduction

Epidermal growth factor receptor (EGFR) is expressed throughout the epidermis [1–3] and is required for key keratinocyte functions, such as proliferation and differentiation. The over-expression of EGFR ligands or upregulation of EGFR is associated with hyperproliferative skin and epithelial squamous cell carcinoma epidermis [4–8]. Conversely, the inhibition of EGFR activity has been shown to impair keratinocyte proliferation, promoting premature terminal differentiation [9]. EGFR also plays a fundamental role during wound healing, through the upregulation of keratinocyte proliferation and migration and EGFR expression is increased following injury [10]. This in turn promotes the re-epithelization of the wound, which is disrupted when EGFR expression is lost [11].

Previous evidence has demonstrated that cooperative signalling between integrins and EGFR can act to enhance cell proliferation, migration and adhesion [12–17]. EGFR has been proposed to form a complex with β1, β3 and β4 integrins during early cell–matrix adhesion, as well as at cell–cell junctions [14,18–21]. Of the potential pathways proposed to act at the nexus of the EGFR-integrin cooperative pathway, focal adhesion kinase (FAK) has been the most widely studied. FAK is a key integrin-dependent kinase and adaptor protein and has been proposed as an important signalling bridge between EGFR and integrins. FAK has previously been shown to form a complex with activated EGFR, potentially via the FAK FERM domain, and this complex is suggested to enhance EGF-stimulated cell migration in fibroblasts [22–25]. Depletion of FAK in mouse epidermis results in epidermal thinning and increased apoptosis [26]. FAK is also implicated in the regulation of cell adhesion and migration, as FAK-deficient cells exhibit increased focal adhesions and decreased directional migration [22,27]. However, the mechanisms that regulate EGFR/FAK-dependent adhesion and migration remain poorly defined.

Our recent discovery of a novel loss-of-function mutation in the EGFR gene (c1428G > A, G428D) that leads to the loss of adhesion in keratinocytes prompted us to study the crosstalk between EGFR and adhesion proteins [28]. Our data reveal that EGFR regulates adhesion dynamics and collective cell migration through the assembly of a complex with FAK, which in turn controls the phosphorylation of ezrin. This results in local suppression of actomyosin contractility at the leading edge of migrating keratinocyte monolayers, enabling the formation of lamellipodia to stimulate forward migration. This work provides insight into how EGFR regulates adhesion signalling and shed light on how disruptions in EGFR signalling lead to the development of skin fragility.

## 2. Material and methods

### 2.1. Plasmids, siRNA, inhibitors and antibodies

eGFP-FAK was kindly provided by Dr Margaret Frame. Ezrin-eGFP was kindly provided by Dr Aleksandar Ivetic ([29], King's College London, London, UK). Talin-eGFP was kindly provided by Dr Gareth Jones. LifeAct-GFP lentivirus was previously published [30]. siR-EGFR-GFP and Y478E and Y478F in ezrin-eGFP were generated by PCR site-directed mutagenesis using the Q5^®^ Site-Directed Mutagenesis kit (New England Biolabs, Ipswich, MA, USA). siEGFR (J-003114–11, Dharmacon, Lafayette, CO, USA) and a non-targeting control siRNA pool were from Dharmacon. AG1478, PP2 and PF228 were all from Tocris (Biotechne, Minneapolis, MN, USA). Primary antibodies for western blotting were mouse monoclonal anti-GAPDH (Chemicom, Mississauga, ON, Canada), anti-GFP (MBL), anti-HSC70 (Sigma Aldrich, St Louis, MO, USA), anti-Src (Millipore, Burlington, MA, USA) and rabbit polyclonal anti-β1 integrin (Millipore, Burlington, MA, USA), anti-EGFR (Cell Signaling, Danvers, MA, USA), anti-Erk1/2 (Cell Signaling, Danvers, MA, USA), anti-ezrin (Biotechne, Minneapolis, MN, USA), anti-FAK (Santa Cruz Biotechnology, Dallas, TX, USA), anti-phospho-EGFR (Y1068, Cell Signaling, Danvers, MA, USA), anti-phospho-EGFR (Y1137, Cell Signaling, Danvers, MA, USA), anti-phospho-Erk1/2 (T202, Y204, Cell Signaling, Danvers, MA, USA), anti-phospho-ezrin (Y478, Abcam, Cambridge, UK; noting the authors performed validation experiments to demonstrate the specificity of this antibody to ezrin; not shown), anti-phospho-FAK (Y397, Cell Signaling, Danvers, MA, USA) and anti-phospho-Src (Y418, Millipore, Burlington, MA, USA). Horseradish peroxidase (HRP)-conjugated secondary antibodies were from Dako (Santa Clara, CA, USA). Primary antibodies for immunostaining: mouse monoclonal anti-EGFR (Santa Cruz Biotechnology, Dallas, TX, USA), anti-E-cadherin (Abcam, Cambridge, UK), anti-vinculin (Sigma Aldrich, St Louis, MO, USA), rabbit polyclonal anti-FAK (Santa Cruz Biotechnology, Dallas, TX, US), anti-phospho-EGFR (Y1068, Abcam, Cambridge, UK), anti-phospho-MLC (S19, Abcam, Cambridge, UK), anti-ezrin (Biotechne, Minneapolis, MN, USA), and anti-β-catenin (Santa Cruz Biotechnology, Dallas, TX, USA).

### 2.2. Cell culture

Normal human keratinocytes (NHK) were maintained in high-glucose DMEM (Sigma Aldrich, St Louis, MO, USA) supplemented with Ham's F12 Nutrient Mixture (Sigma Aldrich, St Louis, MO, US), 10% (v/v) fetal bovine serum (FBS), 2 mM l-glutamine, 1% (v/v) of penicillin–streptomycin (Sigma Aldrich, St Louis, MO, US) 1 x RM+ containing 40 μg ml^−1^ hydrocortisone (Sigma Aldrich, St Louis, MO, USA), 500 μg ml^−1^ insulin (Sigma Aldrich, St Louis, MO, USA), 1 μg ml^−1^ EGF (PeproTech, Rocky Hill, NJ, USA), 0.84 μg ml^−1^ cholera toxin (Sigma Aldrich, St Louis, MO, USA), 500 μg ml^−1^ transferrin (Sigma Aldrich, St Louis, MO, US) and 1.3 μg ml^−1^ lyothyronine (Sigma Aldrich, St Louis, MO, USA). Human embryonic kidney 293T (HEK-293T) cells were maintained in high-glucose DMEM supplemented with 10% (v/v) FBS, 2 mM l-glutamine, 1% (v/v) penicillin-streptomycin. All cell lines were maintained at 37°C in a 5% CO_2_ humidified atmosphere.

### 2.3. Generation of stable cell lines and siRNA

HEK-293T cells were plated to 40–50% confluency the night before transfection. A transfection mixture containing 2.1 μg pCMV8.91, 0.7 μg pMD.G and 3.75 μg of various lentivirus constructs was mixed in 500 μl of OPTIMEM. This was followed by the addition of 22.5 μl of polyethylenimine transfection reagent before incubating for 15 min at room temperature. The mixture was then added to the cells with media containing no antibiotics for 4 h before replacing with OPTIMEM for 48 h. Viruses were then harvested and filtered before adding to NHKs cells containing 8 µg ml^−1^ polybrene. EGFR or control siRNA was mixed with DharmaFECT before adding to cells for 8 h. Media was replaced for 24–48 h before further experiments were performed.

### 2.4. Immunoprecipitation

NHKs stably expressing EGFR-GFP were washed with cold PBS before cold GFP-TRAP lysis buffer (50 mM Tris-HCL pH 7.4, 200 mM NaCl, 2 mM MgCl_2_, 1% (v/v) NP40, 10% (v/v) glycerol, protease inhibitor cocktail set 1, NaF, phosphatase inhibitor) was added to the cells. Cells were scraped and centrifuged to remove cell debris. 1 : 1 of GFP-TRAP_A beads (Chromotek, Munich, Germany) and control agarose resin (Thermo Fisher Scientific, Waltham, MA, USA) were washed thrice with GFP lysis buffer were washed with GFP lysis buffer before cell supernatants were added to the beads and incubated at 4°C for 2 h. Some of the supernatants were also set aside as input. Afterwards, the beads were washed with lysis buffer without detergent before sample buffer-containing dithiothreitol was added to the beads, boiled and analysed by western blotting.

### 2.5. Western blotting

To evaluate protein expression based on their molecular weights, SDS-PAGE was performed using gel with 8–12% (v/v) polyacrylamide resolving layer and a 4% (v/v) stacking layers. The proteins were then transferred to nitrocellulose for 1.5 h at 20 V using a transfer kit (Invitrogen) in Transfer Buffer. The membranes were blocked using blocking buffer (5% (w/v) bovine serum albumin (BSA) in TBS, 0.1% (v/v) Tween) for 1 h at room temperature. This was followed by incubation with appropriate primary antibodies in blocking buffer overnight at 4^◦^C. After membranes were washed with TBST, they were incubated with HRP-conjugated secondary antibodies (Dako, Santa Clara, CA, USA) for 1 h at room temperature. After washing with TBST, proteins were detected ECL chemiluminescence kit (Bio-Rad Laboratories, Hercules, CA, USA) and directly imaged using the BioRad imager digital imaging system. Blots were analysed and processed using BioRad Image Lab.

### 2.6. Phospho-protein profiling

NHKs were subjected to cytoskeleton phospho-antibody array (Full Moon BioSystems, Sunnyvale, CA, USA). Briefly, whole-cell lysates were collected, followed by the labelling of proteins by biotin. The biotinylated proteins were allowed to bind to the pre-blocked microarray slides. The detection of total and phospho-proteins were carried out by the incubation of Cy3-steptavidin. Slides were then transported to Full Moon BioSystems Inc. for scanning and analysis. Background signals were subtracted for each spot before the average median signals were calculated for further analysis. The fold change in phosphorylation was calculated by dividing the intensity of the phospho-protein by the intensity of the total protein. This was then normalized against the control cell line, where 1.5-fold change in phosphorylation was identified as significant.

### 2.7. Cell–matrix adhesion assays

Coverslips were coated with 10 µg ml^−1^ laminin for 1 h at 37°C, and NHKs were plated onto the coverslips for 20, 40 or 60 min before fixation and immunostaining.

### 2.8. Immunofluorescence

Cells were fixed in 4% (v/v) PFA in PBS, pH 7.4 for 15 min on ice for single-cell staining, and in 4% (v/v) PFA in PBS, pH 7.4 with 0.01% (v/v) Triton X-100 for 15 min for monolayer staining. Cells were then permeabilized with 0.1% (v/v) Triton X-100 in PBS before blocking with 5% (w/v) BSA in PBS or TBST for 1 h at room temperature. This was followed by incubation in primary antibodies in blocking buffer at room temperature or at 4°C, before rinsing with PBS or TBST. After incubation with appropriate primary antibodies, secondary fluorescent conjugated antibodies, DAPI and phalloidin (if required) were added for 1 h at room temperature, coverslips were washed and mounted onto slides for subsequent imaging.

### 2.9. Confocal microscopy

Images of fixed cells were taken on a Nikon A1R inverted confocal microscope (Nikon instruments, Melville, NY, USA) with an environmental chamber maintained at 37°C. Images were taken using a 60 × Plan Fluor oil immersion objective (numerical aperture of 1.4). Excitation wavelengths of 488 nm, 561 nm or 640 nm were used. Images were acquired using NIS-Elements imaging software and were processed in Image J. For focal adhesion turnover analysis, cells stably expressing Talin-GFP were taken on a Nikon A1R inverted confocal microscope (Nikon instruments, Melville, NY, USA) with an environmental chamber maintained at 37°C. Images were taken using a 60× Plan Fluor oil immersion objective (numerical aperture of 1.4).

### 2.10. Focal adhesion analysis

To analyse the number and area of focal adhesions at the leading wound edge, cells were fixed and stained with antibody against vinculin to label focal adhesions. A line marking the wound edge was drawn and the length of the wound edge was measured. A region-of-interest extending 20 µm from the wound edge was then selected for focal adhesion analysis. Image was threshold and the number and area of focal adhesions were analysed using ImageJ. For focal adhesion turnover analysis, time-lapse images of 30 s interval were taken and uploaded onto Focal Adhesion Analysis Server (FAAS; https://faas.bme.unc.edu/) to generate adhesion threshold values for subsequent analysis with the detection threshold set as 3.5. The assembly and disassembly rates were obtained through tracking changes in fluorescence intensity from a single adhesion through a different time frame.

### 2.11. Contractility analysis

To determine changes in leading-edge protrusion and contractility, cells were stained with antibody against phospho-MLC and phalloidin. Cells were then scored to determine whether cells contain lamellipodia and/or phospho-MLC band. Phalloidin staining was used as a readout for lamellipodia, where cells with thick actin bundles at the leading edge were not considered to be having lamellipodia. Cells containing a thick phospho-MLC band at the leading cell edge were quantified as a readout for cell contractility.

### 2.12. Widefield time-lapse fluorescence microscopy and protrusion analysis

Cells expressing LifeAct-GFP were imaged using fluorescence time-lapse microscopy on an EVOS FL Auto2 (Thermo Fisher Scientific, Waltham, MA, USA) with an environmental chamber maintained at 37^◦^C. Images were taken using 20 × objective with 3.2 MPCMOS camera. Excitation using GFP LED light cubes were used. Images were acquired using EVOS software and processed using ImageJ where fluorescence images at 0 and 1 h were used for leading-edge protrusion analysis. Images from different time points were first threshold to generate binary image of the leading wound edge. Overlays of images at different time point were then generated where the difference in area can be measured.

### 2.13. Fluorescence recovery after photobleaching

To analyse GFP-FAK turnover, NHKs expressing GFP-FAK was analysed using the NIS-elements advanced research software (Nikon instruments, Melville, NY, USA). ROIs around the focal adhesions were photobleached by a bleach pulse (1 s) at 100% laser intensity at 488 nm. Recovery of fluorescence within the ROI was monitored over 1 min. Background and reference ROIs were selected for background and reference correction. Approximately 20 focal adhesions were averaged to generate one fluorescence recovery after photobleaching (FRAP) curve for a single experiment. The experimental data were fitted using the one-phase decay in GraphPad. Half-life and mobile fraction were then calculated.

### 2.14. Wound healing assays

NHKs were plated in confluency and incubated for 4–24 h in the presence of 2 mM of calcium to promote junction formations. After monolayers were wounded and washed, cell migration into the wound was filmed on an EVOS FL Auto2 (Thermo Fisher Scientific, Waltham, MA, USA) with an environmental chamber maintained at 37°C. Images were taken every hour over 6 h and wound closure was analysed using Image J.

### 2.15. Statistical analysis

Data are represented as mean ± standard error of the mean (s.e.m.). Al statistical tests were carried out using GraphPad Prism (V. 8). The Student's *t*-test was performed for comparing two groups for statistical analysis. Analysis of variance with Tukey's *post hoc* test was used for multiple comparisons. *p* < 0.05 was considered as statistically significant.

## 3. Results

### 3.1. Epidermal growth factor receptor and focal adhesion kinase cooperate to control keratinocyte adhesion

**Figure 1.**
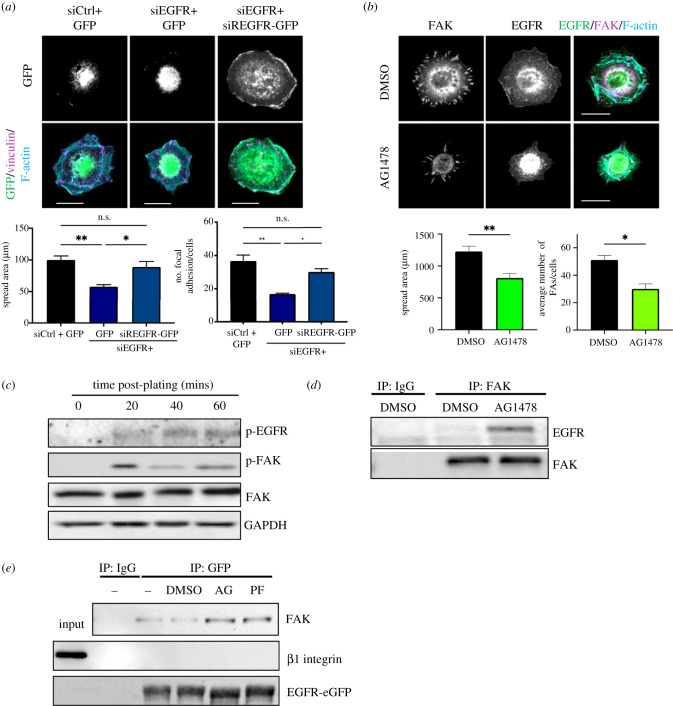
EGFR activity is required for keratinocyte adhesion. (*a*) NHKs stably expressing GFP, siRNA-resistant EGFR-GFP (siR-EGFR-GFP) were transfected with control siRNA (siCtrl) or siRNA targeting EGFR (siEGFR) before plating onto laminin. Cells were left to adhere for 40 min before fixation and immunostaining with vinculin (magenta) and F-actin (cyan). Scale bar 10 µm. Phalloidin was used to quantify the average area per cell. *N* = 3 *p < 0.05, **p < 0.01; (*b*) NHKs were pre-treated with DMSO or EGFR inhibitor (AG1478) for an hour before re-plating onto laminin in the presence of AG1478. After 40 min, cells were fixed and immunostained with FAK (magenta), EGFR (green) and F-actin (cyan). Phalloidin was used to quantify the average area per cell. *N* = 4 *p < 0.05, **p < 0.01; (*c*) NHKs were plated onto laminin before lysates were collected at 0, 20, 40 and 60 min after matrix adhesion. Western blots were probed for phospho-FAK (Y397), FAK, phospho-EGFR (Y1173) and GAPDH. (*d*) Confluent monolayers of NHKs were pre-treated with AG1478 for an hour before wounding. After an additional hour of inhibitor incubation, immunoprecipitation was carried out using an antibody against FAK. Western blot analysis was performed probed with EGFR and FAK. (*e*) Confluent monolayers of NHKs stably expressing eGFP or EGFR-eGFP were pre-treated with AG1478 (AG), PF228 (PF) or DMSO as a negative control. After 1 h, monolayers were wounded and were incubated for an hour with AG1478/PF228 before cell lysates were collected for immunoprecipitation with GFP-TRAP beads. Western blot analysis was carried out probed with FAK, β1 integrin and GFP.

To examine the involvement of EGFR during early keratinocyte adhesion, endogenous EGFR was knocked down in keratinocytes using siRNA (electronic supplementary material, figure S1*a*). EGFR-depleted cells were significantly smaller and had fewer focal adhesion than control transfected cells, and these phenotypes were rescued following re-expression of siRNA-resistant EGFR (siR-EGFR-GFP) (figure 1*a*). To define roles for EGFR kinase activity in this process, cells were treated with the EGFR kinase inhibitor AG1478, resulting in a significant reduction in spread cell area and in FAK-containing focal adhesions at the cell periphery (figure 1*b*). To explore whether EGFR activity was triggered by adhesion in the absence of EGF, cells were plated on laminin for up to 60 min in serum-free media, and the activity of both EGFR and FAK was assessed by western blotting. Data revealed that active EGFR was detectable at 20 min post-plating and that this was coincident with activation of FAK (figure 1*c*) suggesting that both molecules are activated on similar timescales by integrin-dependent adhesion. Notably, however, EGFR and FAK did not colocalize at the periphery of spreading control cells (figure 1*b*), suggesting these kinases are spatially segregated during early adhesion.

Growth of cells in monolayers did not affect basal EGFR or FAK activity, and notably FAK activity was not enhanced in cells treated with EGF for short time periods (electronic supplementary material, figure S1*c*), indicating canonical EGFR activation does not enhance FAK activity. To determine whether localization between EGFR and FAK could be initiated by migratory cues, cells were grown as monolayers, subjected to scratch wounding before co-immunoprecipitation analysis. The result demonstrated an interaction between EGFR and FAK, but only when EGFR or FAK activity was inhibited (figure 1*d,e*). No association between EGFR and β1 integrins was detected under the same conditions (figure 1*f*). These data collectively demonstrate that FAK and EGFR form a complex when both kinases are inactive, and that this complex is not bridged by integrin-FAK binding.

### 3.2. Epidermal growth factor receptor and focal adhesion kinase co-operate to restrict actomyosin and promote focal adhesion dynamics and leading-edge protrusion

**Figure 2.**
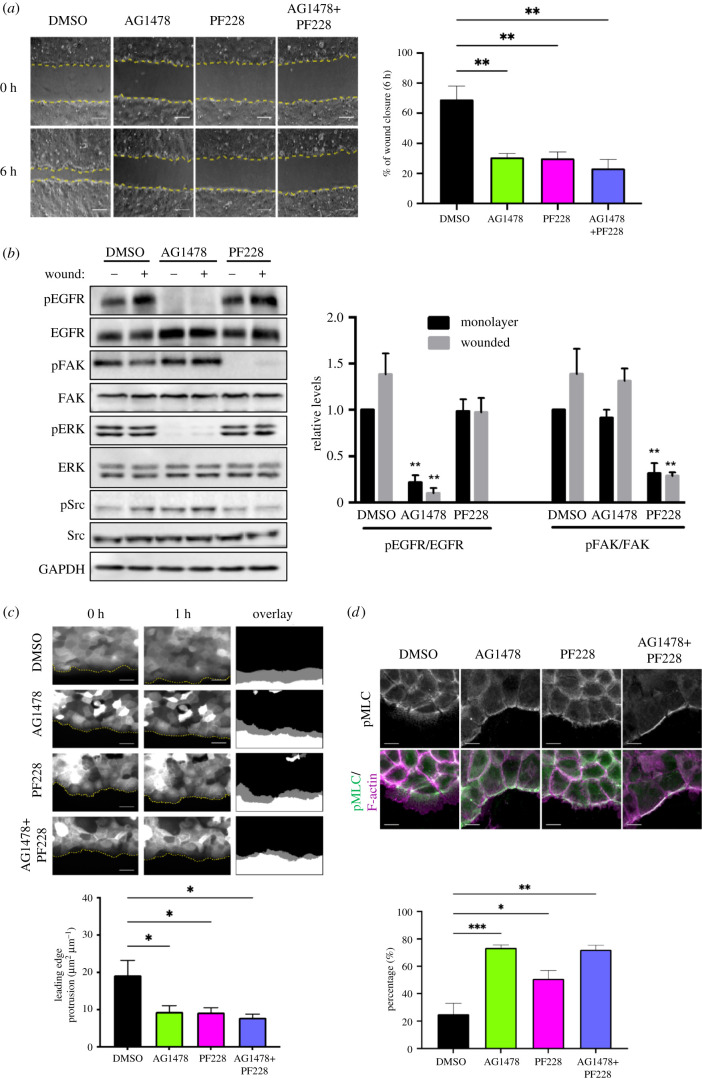
EGFR and FAK cooperate to restrict actomyosin and promote leading-edge protrusion. (*a*) Confluent monolayers of NHKs were pre-treated with EGFR inhibitor (AG1478) and/or FAK inhibitor (PF228) for 1 h before wounding, followed by further treatments with indicated inhibitors before imaging for 6 h. Yellow dashed lines show the leading edge of the wound. Scale bars represent 100 µm. Quantification of the percentage of wound closure after 6 h. *N* = 3 **p < 0.01; (*b*) unwounded or wounded monolayers treated with different inhibitors were subjected for western blotting, probed for phospho-EGFR, EGFR, phospho-FAK, FAK, phospho-Erk1/2, Erk1/2, phospho-Src, Src and GAPDH. Quantitative analysis of band intensities of pEGFR/EGFR and pFAK/FAK was carried out. *N* = 3 **p < 0.01; (*c*) monolayers stably expressing LifeAct-GFP treated with AG1478 and/or PF228 undergoing wound healing were imaged over 1 h. Overlays of the leading edges at the start and end of imaging were generated for the quantification of leading-edge protrusion. Scale bars are 50 µm. *N* = 3 *p < 0.05; (*d*) NHK monolayers were fixed and immunostained with phospho-myosin light chain (pMLC) (green) and F-actin (magenta). Scale bars represent 10 µm. The percentage of cells exhibiting phospho-MLC band at the leading cell edge was quantified. *N* = 3 *p < 0.05, **p < 0.01, *** *p* < 0.001.

To determine whether the spreading defects observed upon EGFR and FAK inhibition translated to a functional outcome, wound healing assays were performed in the presence of FAK inhibitor (PF228) and/or EGFR inhibitor (AG1478). Resulting data demonstrated that treatment with each inhibitor alone resulted in a significant reduction in wound closure compared to DMSO-treated cells, with no additional reduction when both kinases were inhibited (figure 2*a*), suggesting that FAK and EGFR may operate through the same pathway to control collective keratinocyte migration. To determine whether inhibition of EGFR affected the activation of FAK or vice versa, the activity of each kinase was analysed by western blotting in wounded and unwounded cells. Data revealed that each inhibitor was highly effective in reducing the activity of the specific target but had no impact on activity of the other kinase (figure 2*b*), indicating that FAK and EGFR do not regulate phosphorylation of one another.

**Figure 3.**
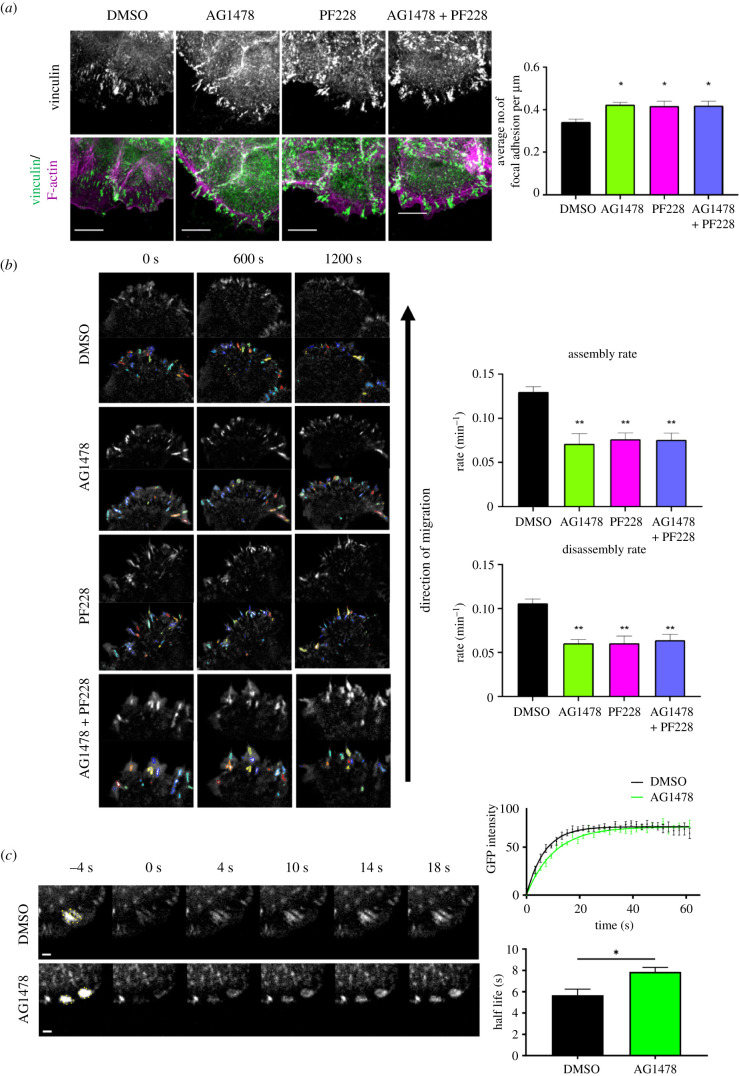
EGFR and FAK suppress focal adhesion dynamics. NHK monolayers treated with EGFR (AG1478) and/or FAK (PF228) inhibitors for an hour before wounding, followed an additional hour of inhibitor incubation. (*a*) Cells were fixed and immunostained with vinculin (green) and F-actin (magenta). Scale bars represent 10 µm. Quantification of the number of focal adhesions normalized to the length of the leading wound edge was carried out. *N* = 3 *p < 0.05; (*b*) monolayers stably expressing talin-GFP were subjected to time-lapse imaging. Coloured outlines indicate focal adhesion detected by the focal adhesion analysis server. Quantification of the rate of focal adhesion assembly and disassembly from 11 to 16 cells/condition from two independent experiments were carried out. **p < 0.01; (*c*) FRAP experiments were carried out where the highlighted regions show the respective bleached regions and their fluorescence recovery after photobleaching. Scale bars represent 2 µm. FRAP recovery curve over time is shown from one representative experiment. Quantification of half-life was carried out using the FRAP recovery curves over time. *N* = 3 *p < 0.05.

To determine the nature of the migration defect upon EGFR and FAK inhibition in more detail, monolayers of LifeAct-GFP expressing cells were imaged for 1 h post-wounding to visualize leading-edge protrusion over time. Analysis of movies demonstrated a significant reduction in protrusion at the leading edge following either EGFR or FAK inhibition, with no further reduction in cells treated with both compounds, again suggesting that EGFR and FAK may operate via a shared same pathway to regulate F-actin-based protrusion at the leading edge (figure 2*c*). Further analysis of fixed cells revealed that both inhibitors also promoted assembly of large actomyosin cables at the leading cell edges that were not present in control cells (figure 2*d*), indicating FAK and EGFR act to suppress contractility at the leading wound edge. We confirmed that these pMLC-positive actin cables were dependent upon RhoA-ROCK signalling, as the ROCK inhibitor Y27632 effectively blocked assembly of the leading-edge actin cables in AG1478/PF228-treated cells (data not shown). To determine whether FAK and EGFR also regulated focal adhesion assembly and dynamics, adhesion markers were analysed in both live and fixed cells. Images of fixed cells stained for vinculin demonstrated a significant increase in the number of focal adhesions in cells along the edge of wounds following EGFR and FAK inhibition (figure 3*a*). This was also seen in mouse keratinocytes treated with EGFR and FAK inhibitors (electronic supplementary material, figure S1*b*). In live cells, assembly and disassembly rates of expressed talin-GFP were also reduced by inhibiting each kinase (figure 3*b*) in agreement with previous studies [31–33]. Combining both inhibitors resulted in a similar increase in focal adhesion number and a comparable reduction in dynamics to similar levels seen in single inhibitor-treated cells (figure 3*a*,*b*). Analysis of GFP-FAK dynamics by fluorescence recovery after photobleaching (FRAP) also revealed that EGFR inhibition significantly slowed FAK recovery within adhesions (figure 3*c*). This suggests that the inhibition of EGFR activity promotes the formation of an FAK/EGFR complex that acts to restrict FAK movement and slow adhesion dynamics.

### 3.3. pY478 ezrin is negatively regulated by focal adhesion kinase/epidermal growth factor receptor

The lack of additive effect of EGFR and FAK co-inhibition on migration, adhesion and actin dynamics suggested that these kinases may act through a common downstream regulator to control these phenotypes. To determine if this was the case, the phosphorylation status of 64 cytoskeletal-associated signalling proteins were quantified in cells treated with siEGFR or FAK/EGFR inhibitors. Resulting data revealed that the depletion and inhibition of EGFR, but not the inhibition of FAK, resulted in a significant reduction in the phosphorylation of Erk1/2, a well-characterized downstream target of EGFR activation, as well as the expected reduction of FAK pY397 levels upon FAK inhibition (electronic supplementary material, figure S2). However, phosphorylation levels of c-Raf, ezrin and Rho/Rac guanine nucleotide exchange factor 2 (RhoGEF2) were significantly increased following EGFR depletion, inhibition or blockade of FAK activity (electronic supplementary material, figure S2) suggesting these common targets may be negatively regulated by FAK and EGFR activity.

**Figure 4.**
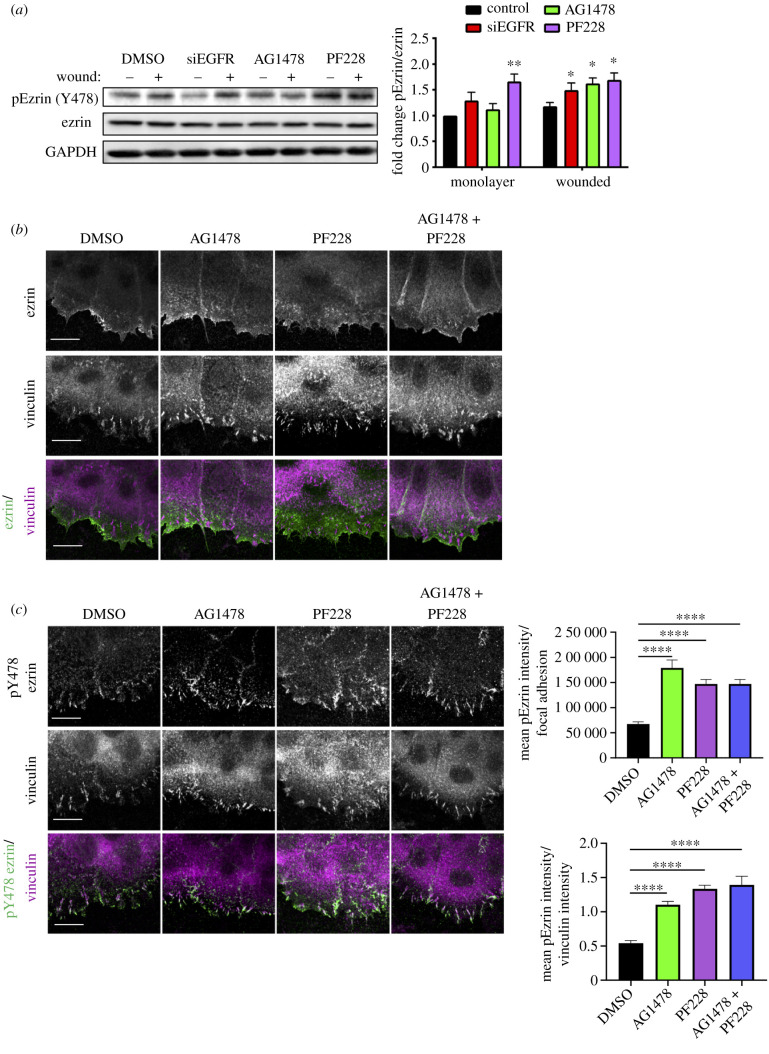
pY478 ezrin is negatively regulated by FAK/EGFR. (*a*) NHKs were transfected with control siRNA (siCtrl) or siRNA targeting EGFR (siEGFR) for 24 h, or treated with EGFR inhibitor (AG1478) or FAK inhibitor (PF228) for 1 h before wounding. Cells were then treated with the various inhibitors for one more hour before further analysis (*a*) western blot analysis, probed for phospho-ezrin, ezrin and GAPDH. Quantification of band intensities of phospho-ezrin over total ezrin was carried out. *N* = 3 *p < 0.05, **p < 0.01; (*b*) confocal images of NHKs monolayers with ezrin (green) and vinculin (magenta). Scale bars represent 10 µm. (*c*) Confocal images of NHK monolayers with phospho-Y478 ezrin (green) and vinculin (magenta). Scale bars represent 10 µm. Quantification of mean pY478 ezrin intensity per focal adhesion and the ratio of mean pY478 ezrin to vinculin intensity are shown from one representative experiment of three experiments. ****p < 0.0001.

Ezrin is a member of the ERM family of proteins that cross-links plasma membrane proteins and the actin cytoskeleton [34]. As such we hypothesized that increased ezrin activation could explain the observed increase in contractility and the decrease in actin dynamics in FAK/EGFR-inhibited cells. Western blotting validated the increased pY478 ezrin levels in FAK- and EGFR-inhibited cells subjected to wounding (figure 4*a*). To further explore the effect of EGFR and FAK on ezrin during collective migration, ezrin localization was analysed in leading-edge migrating cells post-wounding. Images demonstrated that endogenous ezrin localized at the leading cell edge, as well as cell–cell adhesions in control cells with no clear changes following EGFR or FAK inhibition (figure 4*b*). However, pY478 ezrin localized predominantly to vinculin-positive focal adhesions, and a significant increase in pY478 ezrin-containing focal adhesions was seen following EGFR and FAK inhibition (figure 4*c*). These data demonstrate that EGFR and FAK inhibition increase levels of pY478 ezrin within focal adhesions at the leading edge of collectively migrating cells.

### 3.4. Active Src in adhesions is negatively regulated by focal adhesion kinase/epidermal growth factor receptor activity

**Figure 5.**
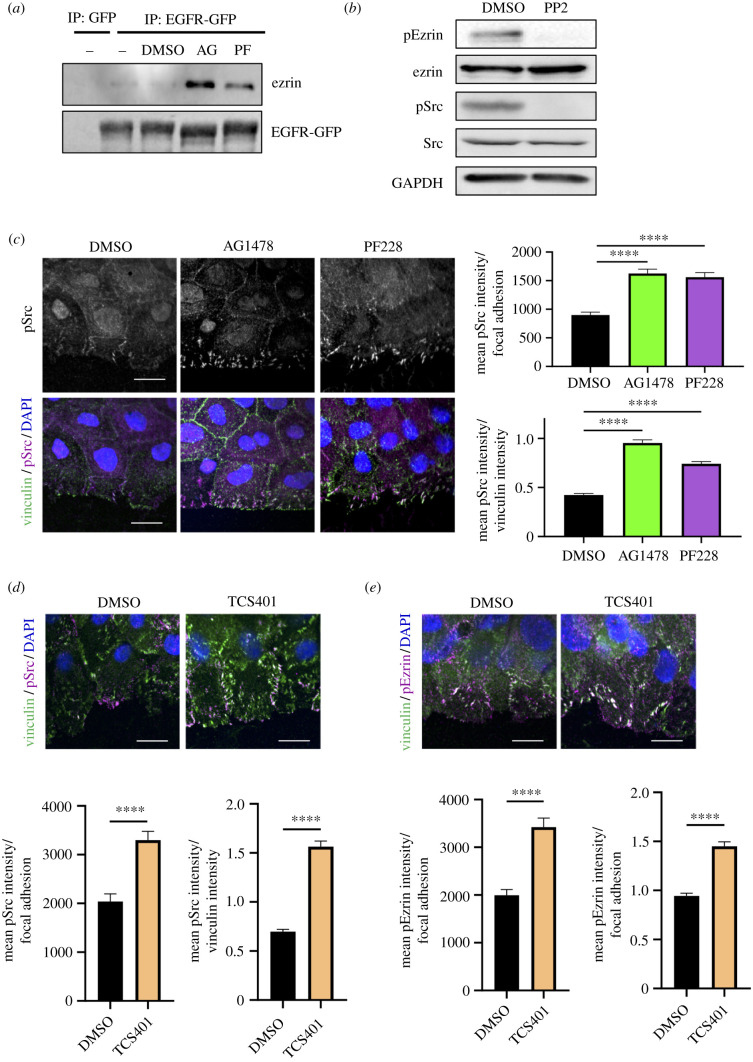
Active Src in adhesions is negatively regulated by FAK/EGFR. (*a*) Monolayers of eGFP or eEGFR-GFP expressing NHKs were treated with EGFR inhibitor (AG1478/AG) and FAK inhibitor (PF228/PF) for 1 h, wounding, followed by immunoprecipitation with GFP-TRAP beads. Representative western blot probed for GFP and ezrin. Note the GFP blot is the same as that shown in figure 1 as target proteins were probed for across all GFP-TRAP experiments for comparison and the blots shown here were from the same experiment as figure 1. (*b*) Monolayers of NHKs were treated with Src inhibitor (PP2) before western blot analysis probed with phospho-ezrin (Y478), ezrin, phospho-Src (Y418), Src and GAPDH. (*c*) NHK monolayers were pre-treated with AG1478 or PF228 before fixation and immunostained for vinculin (green) and pSrc (magenta). Quantification of mean pSrc intensity per focal adhesion and the ratio of mean pSrc intensity to vinculin intensity is shown from one representative experiment of three independent experiments. ****p < 0.0001; (*d*,*e*) NHK monolayers were pre-treated with PTP1B inhibitor (TCS401) for 1 h before wounding. After one additional hour of inhibitor incubation, cells were subjected to fixation and immunostained for vinculin (green) and pSrc (magenta, *d*) or pEzrin (magenta, *e*). Quantification of mean pSrc or pEzrin intensity per focal adhesion and the ratio of mean pSrc or pEzrin to vinculin intensity is shown from one representative experiment of three independent experiments. ****p < 0.0001.

To determine whether enhanced leading-edge localization of pY478 ezrin was coincident with ezrin forming a complex with the previously identified inactive EGFR/FAK complex, EGFR was immunoprecipitated and probed for ezrin. Resulting blots demonstrated that ezrin was indeed part of the EGFR/FAK complex but only when these kinases were inhibited (figure 5*a*) and therefore forming a complex with one another (figure 1*d*–*f*). Src has previously been identified as a kinase responsible for phosphorylating the Y478 site on ezrin [35], and we confirmed this in keratinocytes using the Src inhibitor PP2, that blocked pY478 ezrin (figure 5*b*). Both EGFR and FAK have previously been shown to activate Src [23], and we also demonstrated that added exogenous EGF could stimulate Src activity in keratinocytes (electronic supplementary material, figure S3*a*); however, our western blots and phospho-array analysis showed no significant global change in Src activity upon EGFR/FAK inhibition suggesting basal activity of these kinases does not significantly contribute to Src activity (figure 2*b*; electronic supplementary material, figure S2). We therefore hypothesized that spatial changes to active Src upon EGFR/FAK inhibition may contribute to enhanced pY478 ezrin within adhesions. Indeed, immunostaining revealed that pY418 Src levels were significantly increased at focal adhesions of cells treated with AG1478 or PF228 (figure 5*c*), whereas total levels of pY418 Src remained unchanged in monolayers or single cells (electronic supplementary material, figure S3*a*), consistent with data from our kinase array (electronic supplementary material, figure S2). Moreover, treatment with a Src inhibitor further suppressed collective migration following EGFR/FAK inhibition (electronic supplementary material, figure S3*b*) suggesting Src acts downstream of these kinases via additional pathways. These data indicate that FAK and EGFR restrict active Src localization to adhesions to promote collective cell migration.

EGFR and integrin-based adhesion are known to regulate activity levels of specific phosphatases, in particular SHP2 and PTP1B [36,37]. SHP2 and PTP1B were further investigated to determine whether EGFR/FAK-dependent phosphorylation of both Src and ezrin was dependent on control of these enzymes. EGF stimulation promoted SHP2 activity as previously shown [38]; however, this was not the case in FAK-inhibited cells. Moreover, SHP2 inhibition did not alter ezrin phosphorylation (data not shown), suggesting this phosphatase is not a key player in this pathway. However, acute PTP1B inhibition by treatment with the selective TCS401 compound resulted in significantly enhanced localization of both pSrc and pEzrin to focal adhesions at the leading edge of migrating cells (figure 5*d,e*), very similar to that seen in EGFR- and FAK-inhibited cells (figure 5*c*). This suggests PTP1B is potentially playing a key role in the spatial regulation of these substrates and that the suppression of this phosphatase upon EGFR/FAK inhibition leads to enhanced pSrc and pEzrin specifically to focal adhesions, resulting in defective actin assembly and cell migration.

### 3.5. pY478 ezrin enhances focal adhesion size and leading-edge contractility

**Figure 6.**
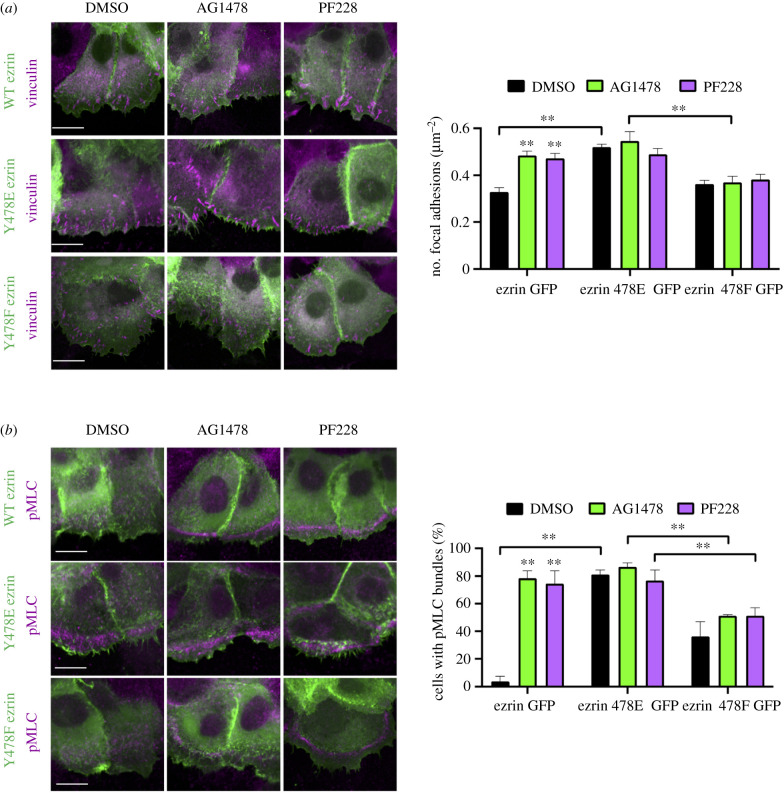
pY478 ezrin enhances focal adhesion size and leading-edge contractility. NHKs stably expressing GFP-tagged wild-type ezrin (WT ezrin), phospho-mimic ezrin-Y478E (Y478E ezrin) or phospho-dead ezrin (Y478F ezrin) were treated with AG1478 or PF228 for 1 h prior to wounding. Afterwards, cells were incubated with the inhibitors for another hour before fixation and immunofluorescence. (*a*) NHKs were immunostained with vinculin (magenta). Scale bars represent 100 µm. Quantification of the average number of focal adhesion over the length of the leading wound edge. *N* = 3 **p < 0.01; (*b*) NHKs were immunostained with phospho-myosin light chain (pMLC) (magenta). Quantification of the average number of cells expressing pMLC bundles at the leading cell edge. *N* = 3 **p < 0.01.

To determine whether the phenotypes induced by EGFR/FAK inhibition were due to enhanced pY478 ezrin, keratinocytes expressing phospho-mimic (Y478E) and phospho-dead (Y478F) ezrin were generated and analysed by microscopy. Images revealed that over-expression of Y478E ezrin resulted in increased focal adhesion number and enhanced leading-edge pMLC, and these phenotypes were not further enhanced by EGFR/FAK inhibition (figure 6*a*,*b*). Y478E ezrin was also strongly localized to the leading edge, coincident with vinculin containing adhesions (figure 6*a*). Conversely, the expression of Y478F ezrin did not enhance focal adhesion formation or pMLC bundle assembly, and these cells also showed no response in either phenotype to FAK/EGFR inhibition (figure 6*a*). Collectively, these data demonstrate that enhanced pY478 ezrin phenocopies EGFR/FAK inhibition, supporting the model whereby EGFR and FAK cooperatively suppress pY478 ezrin levels at leading-edge focal adhesions to enable efficient actin-based protrusion and cell migration.

## 4. Discussion

**Figure 7.**
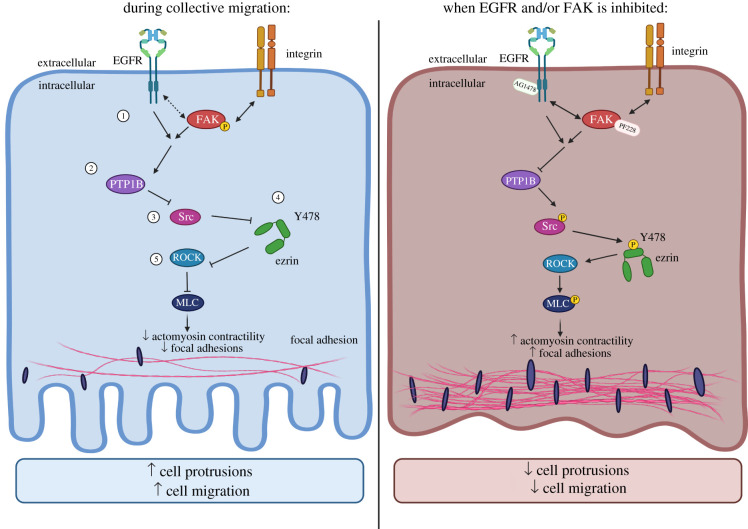
Model of EGFR/FAK-ezrin function in the regulation of cell migration in keratinocytes. During migration, (1) EGFR associates with FAK and ezrin within focal adhesions to regulate their phosphorylation and turnover. EGFR does not appear to be directly associated with integrins. Interactions between EGFR and FAK occur specifically at nascent focal adhesions and vesicles at the leading cell edge. Here, EGFR has been demonstrated to promote the activation of FAK to induce focal adhesion disassembly. FAK is also responsible for the localization of EGFR in focal adhesions. (2) EGFR and FAK together promote the activation of phosphatase PTP1B, which in turn (3) inhibit the phosphorylation and activation of Src. (4) This prevents the phosphorylation of ezrin at Y478, and the subsequent (5) inhibition of ROCK and myosin light chain (MLC). This, in turn, inhibits the actomyosin contractility and focal adhesion turnover to promote forward migration. When EGFR and/or FAK is inhibited, there is an increased association between EGFR and FAK, which in turn inhibits the activity of PTP1B. This is followed by an increased in Src phosphorylation, thus promoting the Y478 phosphorylation of ezrin. This is followed by the activation of ROCK and the subsequent phosphorylation of MLC. A decrease in focal adhesion dynamics and an increased in actomyosin contractility at the leading wound edge result in an inhibition in forward migration.

Here, we have demonstrated that cooperative integrin and EGFR signalling in keratinocytes occurs through the dynamic assembly of an EGFR/FAK complex, which in turn inhibits ezrin phosphorylation through the activation of PTP1B and inactivation of Src (figure 7). This allows actin filaments to re-organize to form lamellipodia, promoting forward migration. EGFR and FAK form a complex with each other, but not with β1 integrins, consistent with previous reports [22,39]. However, our data show the inactivation of FAK results in inactive EGFR recruitment to focal adhesions, without changing the overall EGFR phosphorylation. Moreover, a reduction in FAK turnover at focal adhesions at the leading edge occurs in EGFR-inhibited cells, suggesting EGFR promotes activation of FAK to enhance focal adhesion disassembly and cell migration. The results observed here agree with previous studies showing that active EGFR promotes disassembly of focal adhesions [31] and defects in focal adhesion turnover occur in FAK-defective cells [27,32,33]. The inhibition of FAK and EGFR also causes an accumulation of actin bundles, inhibiting membrane protrusion, correlating with previous studies where an increase in F-actin was observed in EGFR-inhibited cells [40] and larger F-actin cables seen in FAK-knockdown mouse keratinocytes [33]. It should be noted that our findings in increased myosin bundles seem inconsistent with previous findings [17,41]. However, the previous reports focus on the contributions of EGFR in early cell spreading events, rather than the collective cell migratory behaviour demonstrated in this study. Therefore, it is likely that different EGFR-dependent mechanism was involved in the regulation of myosin contractility during collective cell migration. It is currently unclear how inactive EGFR is relocated to the focal adhesion-containing FAK at the leading edge. One possibility is the inhibition of FAK results in the sequestration of membrane-associated inactive monomeric EGFR to sites of F-actin bundling, leading to the formation of the inactive EGFR/FAK complex. This may represent a mechanism for these kinases to slow adhesion dynamics and migration under low EGF conditions, where both kinases are inactive. Future studies analysing local dynamics and dimerization status of EGFR upon FAK inhibition will provide further insight into how these complexes may assemble at the leading edge.

Our data show that inactive FAK binds preferentially to EGFR. Although FAK has been widely studied as a modulator of migration in its active form, is also notable that inactive FAK can act as a binding partner and scaffold for other proteins involved in migration. One example is the Arp2/3 complex that binds to inactive FAK at the leading edge of motile cells [42]. Inactive FAK can also bind to the dynein-associated protein Nudel and in doing so, displaces Nudel from paxillin and modulates cell–matrix adhesion. The formation of the FAK/EGFR complex may therefore represent a new non-catalytic role for FAK in coordinating EGFR signalling and provide a mechanism for epithelial cells to actively suppress migration when EGFR and FAK activity is low.

In this study, we have also identified ezrin as a novel shared target for EGFR and FAK. We show pY478 ezrin forms a complex with EGFR/FAK and suppresses focal adhesion dynamics and migration, which has been shown to also occur in ezrin-depleted cells [43]. The Y478 residue is not a canonical phosphorylation site, such as T567 and Y353, that are required for the conformational activation of ezrin. Our data would suggest pY478 ezrin can actively recruit and enhance F-actin bundles and actomyosin contractility proximal to focal adhesions. It is currently unclear whether the phosphorylation at Y478 may stabilize the open conformation of ezrin, and whether this modification results in ezrin association with proteins that control F-actin assembly. We have further shown Y478 phosphorylation is promoted by the activation of Src within focal adhesions, in agreement with a previous study showing this site to be substrate for Src kinases [35]. Moreover, the inhibition of PTP1B results in an increase in active Src and pY478 ezrin within focal adhesions, phenocopying the effects of EGFR/FAK inhibition. This suggests inactive FAK/EGFR complexes suppress local PTP1B activation and in doing so, enhance active Src. It is notable that we also identified RhoGEF2 as significantly activated in EGFR/FAK-inhibited cells. This may represent an interesting target for future studies to determine contributions to Rho-induced actomyosin assembly at the leading edge.

Previous studies have shown that both EGFR and FAK can activate Src, and indeed, we saw robust increased Src activity in EGF treated keratinocytes in our study, but no change in global Src when EGF or FAK were inhibited. One potential explanation for this apparent discrepancy lies in the basal activity state of EGFR/FAK in keratinocyte monolayers. Many of the previous studies have been performed in cancer cells that are highly motile and display increased activity of these kinases, meaning they may positively contribute to Src activity, whereas alternative pathways regulate basal Src activity in keratinocytes. Moreover, most studies analyse bulk pSrc levels biochemically, rather than by imaging. Our study shows pSrc is only elevated in focal adhesions upon EGFR/FAK inhibition, suggesting this is a tightly spatially controlled event.

EGFR is constitutively expressed in normal skin and hair follicles and is involved in mediating a wide range of processes in keratinocytes, such as differentiation and proliferation [2,3,44], as well as in many pathophysiological conditions, during wounding and injury [45]. Therefore, disruptions to EGFR-dependent pathways often lead to the development of skin fragility diseases or epithelial cancers and therefore EGFR has been a popular target for cancer. However, cancer patients receiving EGFR inhibitors often develop eventual resistance to the treatments. Emerging evidence has proposed that EGFR TKI resistance operates through an integrin-mediated pathway [46,47], with some showing the increased involvement of FAK in the resistance of cancer cells to EGFR TKIs [48,49]. The treatment with erlotinib and FAK inhibitors together in EGFR TKI-resistant NSCLC cells has been shown to effectively reduce cell viability [50]. Here, we describe a novel mode of cooperation of EGFR and FAK signalling, which converge on ezrin, and has provided the opportunity to investigate possible treatments such as targeting FAK and/or ezrin in combination with EGFR to overcome EGFR TKI resistance in cancer cells.

In summary, we have identified a new role for inactive FAK/EGFR complexes in focal adhesions in promoting local phosphorylation of Src, leading to phosphorylation of ezrin at Y478 in a PTP1B-dependent manner. pEzrin at focal adhesions promotes spatial assembly of actomyosin cables that restrict forward migration. Our data provide new insight into how active and inactive pools of these key regulatory kinases cooperate to control spatial signalling events to control epithelial cell adhesion and migration.

## Data accessibility

Data and materials are available from the authors upon request.
